# Lack of robust evidence for a *Wolbachia* infection in *Anopheles gambiae* from Burkina Faso

**DOI:** 10.1111/mve.12601

**Published:** 2022-07-25

**Authors:** Simon P. Sawadogo, Didier A. Kabore, Ezechiel B. Tibiri, Angela Hughes, Olivier Gnankine, Shannon Quek, Abdoulaye Diabaté, Hilary Ranson, Grant L. Hughes, Roch K. Dabiré

**Affiliations:** ^1^ Département de Biologie Médicale et Santé Publique, Institut de Recherche en Sciences de la Santé Bobo‐Dioulasso Burkina Faso; ^2^ Département de Virologie et de Biotechnologies Végétales, Institut de l'Environnement et de Recherches Agricoles (INERA) Ouagadougou Burkina Faso; ^3^ Department of Vector Biology Liverpool School of Tropical Medicine Liverpool UK; ^4^ Département de Biologie et de Physiologie Animales, Université Joseph K‐Zerbo Ouagadougou Burkina Faso; ^5^ Departments of Vector Biology and Tropical Disease Biology, Center for Neglected Tropical Disease Liverpool School of Tropical Medicine Liverpool UK

**Keywords:** *Anopheles gambiae*, *biocontrol*, *native infection*, *population replacement*, *population suppression*, *w*Anga, *Wolbachia*

## Abstract

The endosymbiont *Wolbachia* can have major effects on the reproductive fitness, and vectorial capacity of host insects and may provide new avenues to control mosquito‐borne pathogens. *Anopheles gambiae* s.l is the major vector of malaria in Africa but the use of *Wolbachia* in this species has been limited by challenges in establishing stable transinfected lines and uncertainty around native infections. High frequencies of infection of *Wolbachia* have been previously reported in *An*. *gambiae* collected from the Valle du Kou region of Burkina Faso in 2011 and 2014. Here, we re‐evaluated the occurrence of *Wolbachia* in natural samples, collected from Valle du Kou over a 12‐year time span, and in addition, expanded sampling to other sites in Burkina Faso. Our results showed that, in contrast to earlier reports, *Wolbachia* is present at an extremely low prevalence in natural population of *An*. *gambiae*. From 5341 samples analysed, only 29 were positive for *Wolbachia* by nested PCR representing 0.54% of prevalence. No positive samples were found with regular PCR. Phylogenetic analysis of 16S rRNA gene amplicons clustered across supergroup B, with some having similarity to sequences previously found in *Anopheles* from Burkina Faso. However, we cannot discount the possibility that the amplicon positive samples we detected were due to environmental contamination or were false positives. Regardless, the lack of a prominent native infection in *An*. *gambiae* s.l. is encouraging for applications utilizing *Wolbachia* transinfected mosquitoes for malaria control.

## Graphical abstract

- The natural infection of endosymbiont *Wolbachia* was re‐evaluate in *Anopheles gambiae* s.l. from Burkina Faso.
- *Wolbachia* is present at an extremely low prevalence in natural population of *An. gambiae* s.l.
- Regardless, the lack of a prominent native infection in An. gambiae s.l. is encouraging for applications utilising Wolbachia transinfected mosquitoes for malaria control.

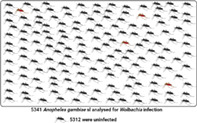

## BACKGROUND

*Wolbachia* is an obligate intracellular bacterial symbiont, found in many insect species that, in recent years, has shown great potential for use in vector‐borne pathogen control. It is well known for its ability to manipulate host reproduction enabling it to spread into insect populations (Werren et al., 2008). Additionally, *Wolbachia* can inhibit the development of diverse pathogens (Hedges et al., 2008; Hughes et al., 2011; Kambris et al., 2009; Kambris et al., 2010; Moreira et al., 2009; Shaw et al., 2016) which makes *Wolbachia* an attractive agent for pathogen control. Some strains of *Wolbachia* protect insect hosts from viral infections (Chrostek et al., 2013; Hedges et al., 2008; Glaser & Meola, 2010) and the presence of *Wolbachia* in *Aedes aegypti* mosquitoes impair infections with dengue and other arboviruses (Moreira et al., 2009; Walker et al., 2011). Based on these findings, releases of *Wolbachia* infected male and female mosquitoes have been undertaken with the aim of spreading *Wolbachia*‐mediated resistance to viruses in natural mosquito populations (Hoffmann et al., 2011) with initial clinical trials showing positive outcomes (Indriani et al., 2020; Utarini et al., 2021). In addition to population replacement approaches, *Wolbachia* has also been exploited for population suppression control strategies whereby infected males are released to reduce mosquito numbers by inducing cytoplasmic incompatibility when mating with uninfected females (Crawford et al., 2020; LAVEN, 1967; Zheng et al., 2019).

In *Anopheles* mosquitoes, there are several reports indicating amplification of the *Wolbachia* 16S rRNA gene fragment by nested PCR. In general, these studies find *Wolbachia* PCR positive individuals at low frequency in the population (Ayala et al., 2019; Baldini et al., 2014; Gomes et al., 2017; Shaw et al., 2016). Some studies have reported a negative correlation between amplicon positive mosquitoes and *Plasmodium* development in natural populations (Gomes et al., 2017; Shaw et al., 2016). Recently, it has been shown that *Anopheles moucheti* and *An*. *demeilloni* possess high density infections with *Wolbachia* observed in the germline, and complete genomes recovered (Jeffries et al., 2018; Quek et al., 2022; Walker et al., 2021). Significant efforts to establish artificially transinfected lines of *Anopheles* with *Wolbachia* have proved largely unsuccessful (Walker et al., 2011). Importantly, a stable line was established in *An*. *stephensi*, a vector of malaria in southern Asia, using *Wolbachia* from *Ae*. *albopictus* (*w*AlbB), which conferred resistance to *Plasmodium falciparum* infection (Bian et al., 2013). However, the *Wolbachia* infection induced fitness costs on the host, which would likely prevent establishment of the bacterium in mosquito populations (Joshi et al., 2014). Somatic, transient infections of the *Wolbachia* in *An*. *gambiae* were shown to significantly inhibit *P*. *falciparum* (Hughes et al., 2011), but the interference phenotype is variable with other *Wolbachia* strain‐parasite combinations (Hughes et al., 2012, 2014; Murdock et al., 2014). Interestingly, *Wolbachia* and other gut‐associated microbes have negative associations (Hughes et al., 2014; Rossi et al., 2015; Zink et al., 2015), and these microbial interactions affect the biology of the host and transmission of the bacterium (Hughes et al., 2014), offering a possible reason for the lack of infection in some *Anopheles* species.

Despite the widespread report of amplification of *Wolbachia* by PCR in *Anopheles*, there is conjecture in the literature if *Anopheles* mosquitoes are truly infected with *Wolbachia* (Chrostek & Gerth, 2019). Most of the evidence stems from nested PCR approaches, a technique that is highly sensitive. This has led to suggestions that nested PCR may detect environmental *Wolbachia* DNA that the mosquito has encountered (Chrostek & Gerth, 2019). The low density and prevalence of infection and the phylogenetic diversity of strains reported indicate the infection is not stable in these associations or environmental DNA is being amplified. It is imperative to verify the prevalence of natural infections, and identify any native strains of *Wolbachia* in *Anopheles*, as these infections could impede population suppression or replacement control approaches exploiting transinfected lines. We previously reported high levels of natural infection in *An*. *gambiae* from Burkina Faso (Baldini et al., 2014; Shaw et al., 2016), but here, we re‐evaluate *An*. *gambiae* mosquitoes from these sites to examine the prevalence of infection in a wider geographical and temporal sample set. Surprisingly, we find low levels of amplicon positive mosquitoes, calling into question the findings from our earlier study.

## METHODS

### Study area and mosquito collection

**FIGURE 1:**
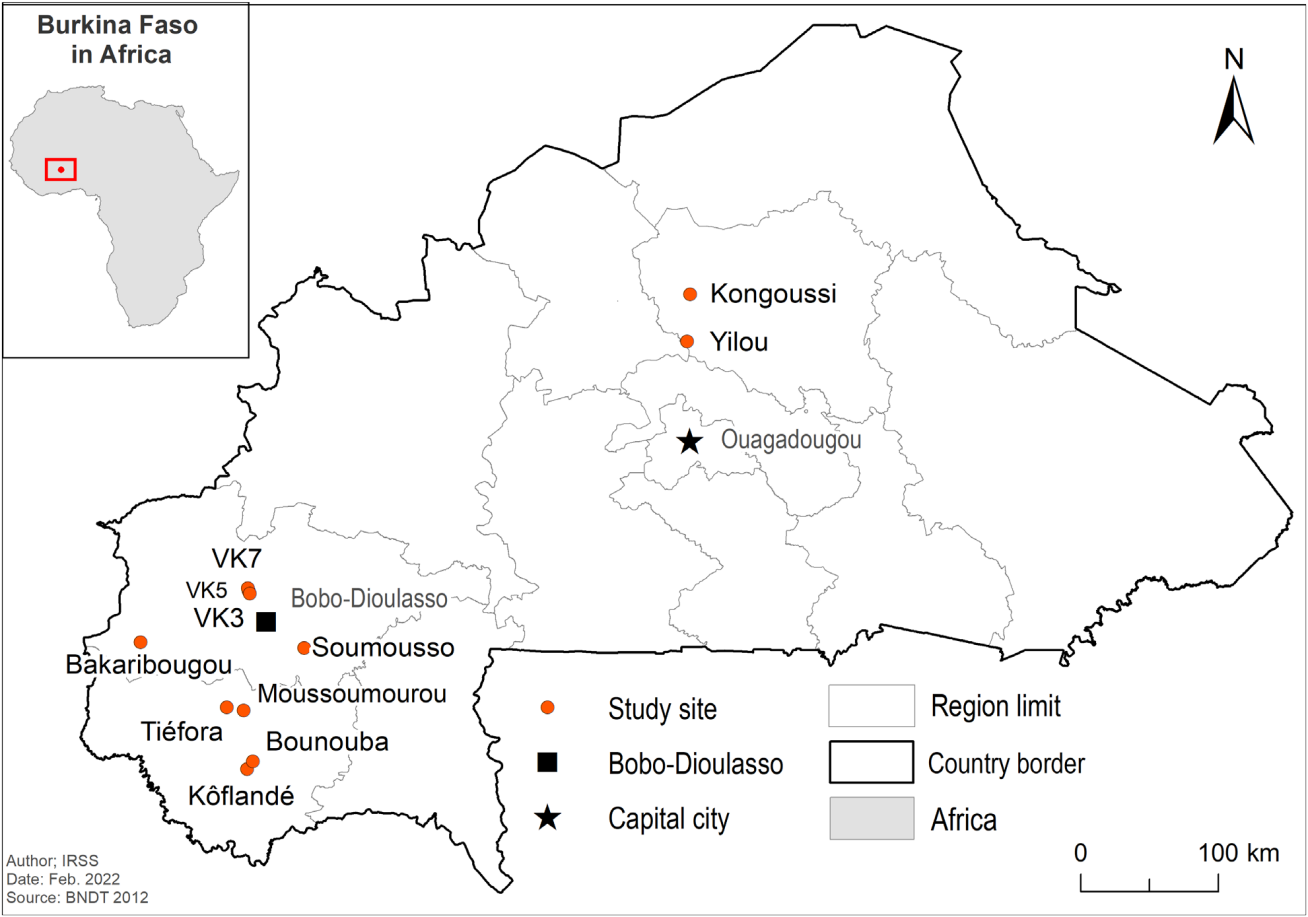
Map of Burkina Faso showing mosquito sampling sites

*Anopheles gambiae s*.*l*. mosquitoes were collected in seven villages during the rainy seasons of 2006, 2011, 2012, 2015, 2016 and 2018. These sites are located in the Sahelian zone (Kongoussi and Yilou) and Sudan‐savanna zone (VK3, VK5, VK7, Soumousso and Tiefora) (Figure 1) of Burkina Faso. Adult mosquitoes were collected from the resting sites (inhabited houses, uninhabited houses, wood piles and clay pots) using mechanical aspirators or CDC light traps; males were also collected from mating swarms using an insect net (Diabaté et al., 2006). Adults were morphologically identified using the standard taxonomic key (Gillies & Coetzee, 1987; Gillies & de Meillon, 1968). Samples were stored at −20°C prior to molecular analyses.

### Mosquitoes DNA extraction and molecular analysis

DNA was extracted from 4953 *An gambiae* s.l. mosquitoes using the CTAB 2% extraction method. PCR was used to identify the species, as described previously (Santolamazza et al., 2008). To screen for *Wolbachia*, conventional (PCR using *Wolbachia* specific primers [Wspecf/Wspecr] was used to amplify the 16S rRNA gene Werren & Windsor, 2000). All samples were also analysed by nested PCR using the primers we used previously (Shaw et al., 2016). Amplicon positive samples were repeated using the nested PCR to confirm infection and the PCR products purified by filtration using NucleoFast 96 PCR DNA purification plate. For Sanger sequencing, positive (DNA from *Culex* mosquitoes collected in the city of Bobo‐Dioulasso) and negative controls were included to rule out PCR contamination. Samples were sequenced by GenoScreen.

In parallel to the 4953 samples, we analysed a total of 388 samples, all collected as larvae either from VK7 (*n* = 268) or from the Cascades district (*n* = 120) were analysed at LSTM. These samples were collected between October 2011 and September 2014 and included 292 samples that had been exposed to deltamethrin (199 survivors, 93 dead) (see Data S1). All were tested using the 16S‐ Wspecf/Wspecr primers (Werren & Windsor, 2000).

### Phylogenetic analysis

Phylogenetic analyses of *Wolbachia* samples were performed on the conserved 16S rRNA sequences. Accession numbers of the sequences obtained in the present study and used to construct the tree are listed in Table 1. Contigs obtained were cleaned and assembled de novo using Geneious v. 8.1.7 (Biomatters Ltd). All sequences were subjected to BLAST search tools in NCBI using Geneious and subsequently to pairwise sequence comparison (Altschup et al., 1990; Bao et al., 2014). The homologous sequences were retrieved for phylogenetic analysis based on BLAST results.

**TABLE 1:** Spatial and temporal variations of *Wolbachia* infection prevalence in Burkina Faso

Year	Village	Species	Tested	Amplicons negative	Amplicons positive (first nested PCR)	Amplicons positive (repeated nested PCR)
2006	Soumousso	*An*. *gambiae*	331	310	24 (7.25%)	21 (6.34%)
	VK7	*An*. *coluzzii*	176	170	19 (11.18%)	6 (3.41%)
2011	VK3	*An*. *coluzzii*	506	506	0	0
2015	Soumousso	*An*. *gambiae*	20	20	0	0
2016	Soumousso	*An*. *gambiae*	562	562	0	0
	VK5	*An*. *coluzzii*	868	867	1 (0.16%)	0
2018	Kongoussi	*An*. *coluzzii*	3	3	0	0
	Soumousso	*An*. *gambiae*	237	237	0	0
	VK3	*An*. *coluzzii*	132	130	2 (1.52%)	2 (1.52%)
	VK5	*An*. *coluzzii*	853	853	0	0
	VK7	*An*. *coluzzii*	1200	1200	0	0
	Yilou	*An*. *coluzzii*	65	65	0	0
	*Total*		*4953*	*4923*	*46 (0*.*93%)*	*29 (0*.*59%)*

From this, we obtained a total of 70 sequences of 16S RNA from *Wolbachia* of supergroups A and B. This number also included 13 sequences that were reportedly isolated from *An*. *gambiae*. These 70 sequences were added to the 17 sequences obtained from this study, as well as an additional sequence from *Wolbachia* of *Culex* mosquitoes, sequenced as part of this study. All of these 88 sequences were then used as input into the program MAFFT v7.455 (Katoh and Standley, 2013), and aligned using default parameters. The resultant alignment was then manually processed to remove ambiguously aligned regions at the 5′ and 3′ ends, as well as any columns that contained undetermined nucleotides from sequencing. This curated alignment was then used as input into the program IQTree v1.6.1 (Nguyen et al., 2015) to build a phylogenetic tree, utilizing the DNA substitution model K2P + G4 (best‐fit model as determined by the ModelFinder algorithm, Kalyaanamoorthy et al., 2017), with 1000 non‐parametric bootstrap replicates. The tree was then visualized and edited using FigTree v.1.4.4.

## RESULTS

No amplicon positive samples were found with the regular PCR but a total of 46 of the 4953 amplicons were positive for *Wolbachia* using the more sensitive nested PCR representing a prevalence of 0.93%. The nested PCR was repeated for each of the initial 46 positive mosquitoes and 29 remained positive on the repeat PCR, representing a prevalence of 0.59% (29/4953). The inability to confirm 17 individuals in a repeated nested‐PCR suggested the template is at the limits of detection in these samples.

*Wolbachia* was only detected in samples from two of the six years; in 2006, *Wolbachia* prevalence was 5.33% (*n* = 507) and 0.08% (*n* = 2490) in 2018 (Table 1). Regarding the spatial distribution, all positive specimens were found in just three of the seven villages (VK3, VK7 and Soumoussso), all located in the Sudan savanna zone in the western region of Burkina Faso. The prevalence of positive samples was low with 0.31% (2/638) in VK3, 0.36% (6/1376) in VK7 and 1.87%: (21/1150) in Soumousso. A significantly higher proportion of *An*. *gambiae* s.s was amplicon positive for *Wolbachia* than *An*. *coluzzii* (χ^2^ = 23.493, df = 1, *p* < 0.0001; *An*. *gambiae* 2.08% (24/1150); *An*. *coluzzii* 0.53% (22/4151)).

Concerning the 388 samples analysed in LSTM from VK7 and Tiefora, no positive amplicon was found in any sample. However, positives bands were observed in positive control using the same protocol.

### *Phylogenetic analysis of* Wolbachia

**FIGURE 2:**
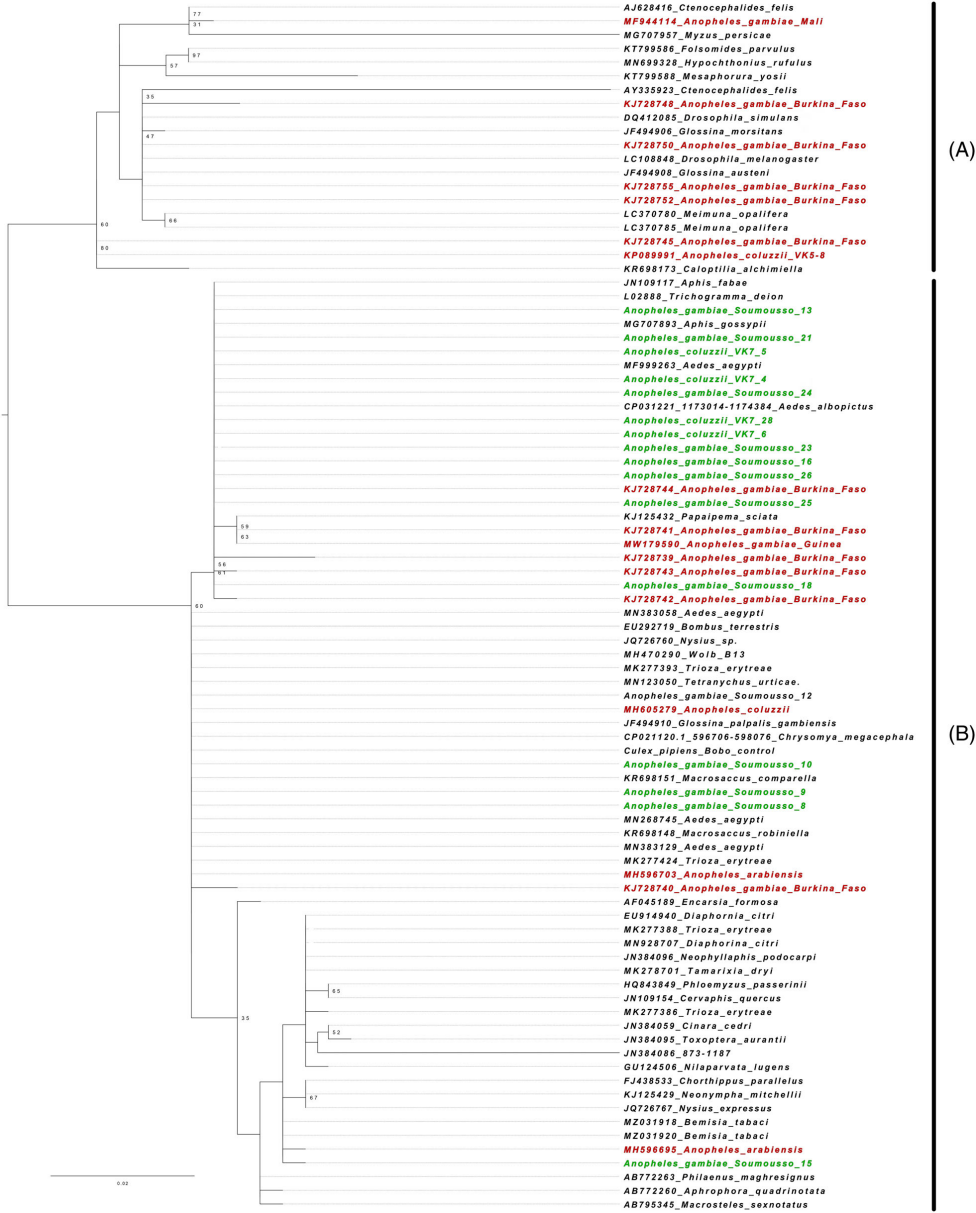
Phylogenetic analysis of *Wolbachia* strain. The green colour represents the sequences found in our samples, whilst red colour represents sequences previously identified in *Anopheles gambiae*

Phylogenetic analysis of the 16S rRNA gene sequences used in this study (70 published sequences, 17 from this study, one positive control from *Wolbachia* of *Culex pipiens*) resulted in a curated alignment of 317 nucleotides in length. A midpoint‐rooted tree showed that all sequences obtained from this study clustered with supergroup B *Wolbachia*. From this study, 12 sequences of *An*. *gambiae* and *An*. *coluzzii* were noted to cluster into a weakly supported clade (Figure 2) alongside *w*AlbB, and six previously published sequences collected from *An*. *gambiae* in Burkina Faso and Guinea. The remaining six sequences obtained from this study were observed to be distributed throughout supergoup B, with none in supergoup A. Contrasting with the sequences from this study, previously published sequences from *An*. *gambiae* were observed to be distributed throughout both supergroups A and B (Figure 2).

When the alignment was reduced to look at the 18 sequences from this study, a total of six variant sites were observed, with five of these sites being unique to one sequence (Data S2, sample Sawadogo‐15). Expanding this alignment to include all sequences from *Anopheles* mosquitoes, as well as *Wolbachia* from *A*. *albopictus*, a total of 31 variant sites were observed. The majority of these variant sites were observed to occur within seven of the published *Wolbachia* sequences, all of which cluster within supergroup A of the phylogenetic tree.

## DISCUSSION

Despite being common among *Culex* and some *Aedes* mosquitoes (Carvajal et al., 2019), *Wolbachia* infections are relatively rare in most *Anopheles species*. While there are emerging reports suggesting that some *Anopheles* possess low prevalence infections, these studies use nested PCR which is a highly sensitive technique that could amplify environmental DNA. As such the veracity of these studies has been questioned (Chrostek & Gerth, 2019). In contrast, a recent study demonstrated that *An*. *moucheti* and *An*. *demeilloni* possessed high‐density maternally transmitted *Wolbachia* strains (Walker et al., 2021). Further genomic analysis of these strains indicated similar metabolic pathways to other *Wolbachia* strain and a close relationship to *Wolbachia* strains found to stably infect *Drosophila simulans* and *D*. *mauritiana* (Quek et al., 2022). Our expanded survey of *Wolbachia* from *An*. *gambiae* collected in Burkina Faso found an extremely low prevalence of amplicon positive samples using the nested‐PCR screening approach.

In our study, analysis of 4953 samples collected over a twelve‐year period in Burkina Faso found only 0.59% (29/4953) were amplicon positive for *Wolbachia*. This contrasts with an infection rate of 46% (275/602) in *An*. *coluzzii* in 2014 in the same area (Shaw et al., 2016) and varying spatially and temporally with samples from Mali with a minimal prevalence of 45% in Dangassa in 2015 reaching 95% (38/40) in *An*. *gambiae* sl in Kenieroba in 2016 (Gomes et al., 2017). In our study, screening conducted in parallel at the Liverpool School of Tropical Medicine, to detect *Wolbachia* in *An*. *gambiae* sl. survivors of pyrethroid exposure collected in 2011 and 2012 from the same geographic region, failed to detect any *Wolbachia* samples in 348 individuals screened. Corroborating our findings, more recent studies using amplicon sequencing of the 16S rRNA gene also found minimal evidence for *Wolbachia* infection of *An*. *gambiae* in Burkina Faso. The relative abundance of *Wolbachia* in the *An*. *gambiae* microbiome was 0.00002% (Zoure et al., 2019), while amplicon sequencing of nested‐PCR positive individuals only found 42 *Wolbachia* reads constituting 0.04% relative abundance of the microbiome (Straub et al., 2020). Taken together, these data indicate that *An*. *gambiae* are free from *Wolbachia* or if these are true infections, their low prevalence in the population suggests it is challenging for the bacterium to establish within the population.

While some of our amplicons were Sanger sequenced and had similarity to *Wolbachia*, we cannot discount the possibility these were false positives. While we did not explicitly determine the specificity rate of this nested PCR, presuming it had a specificity rate of 99%, we would expect on average 49 false positive PCRs. These figures are in line with our 29 amplicon positive samples. Similarly, these amplicon positive samples, several of which appear to be on the limits of detection, could have been due to amplification of environmental DNA encoding for the *wsp* gene. For example, it is known that microbial DNA can persist within soil for years (Nielsen et al., 2007). It is also possible that the signal was due to *Wolbachia* bacteria that were transiently associated with the mosquito. This may be facilitated by ectoparasitic mites or midges, infection with endoparasitic nematodes, cohabitation with other arthropods hosting these bacteria, or acquired through the nectar feeding in plants (Chrostek et al., 2017). Indeed, it has been shown that *Wolbachia* can persist in plants on which *Wolbachia*‐infected insects feed and then be detected in previously uninfected insects reared on the same plants (Chrostek et al., 2017). As malaria vectors feed on plant nectar and fruits in the wild, traces of environmental DNA encoding for *Wolbachia* genes may accumulate in their guts (Chrostek & Gerth, 2019). It is not currently possible to say with certainty if any of the above possible sources of potential contamination may have contributed to the high prevalence we observed in the 2014 study. However, our current study shows that *Wolbachia* is present at a very low prevalence in *An*. *gambiae*, we saw more amplicon positive samples from villages (Soumousso (6.34%) and VK7 (3.41%)) where it had been detected previously in 2006 (Baldini et al., 2014; Shaw et al., 2016). This could possibly indicate there was a biological factor, such as a *Wolbachia*‐infected arthropod that cohabitated with *An*. *gambiae*, in this region and time, producing amplicon positive signals. As low prevalence, low intensity infections are challenging to accurately detect it is not possible to conclude with certainty that the *Wolbachia* detected in this study, or our earlier published studies (Baldini et al., 2014; Shaw et al., 2016) are native infections of *Anopheles*.

The evidence suggests there is no established *Wolbachia* infection in *An*. *gambiae* in Burkina Faso. These mosquitoes therefore appear to be an open niche for transinfection (Hughes & Rasgon, 2014). Although challenging, the development of a transinfected *An*. *gambiae* line would enhance prospects to implement either *Wolbachia*‐based population suppression or population replacement control strategies. While improbable, should it eventuate that there is a stable low prevalence *Wolbachia* infection that induces reproductive manipulations in *An*. *gambiae*, control approaches can still be implemented, as *Wolbachia* control can still be undertaken in species which harbour native infections (Jeffries & Walker, 2016). For example, a double infection could be used for population replacement while a strain that induces bidirectional cytoplasmic incompatibility would be effective for population suppression control. As such, the pursuit to develop a stable transinfected line in *An*. *gambiae* is a worthwhile endeavour to expand the tools available for the control of malaria.

## AUTHOR CONTRIBUTIONS

Simon P. Sawadogo, Grant L. Hughes, and Roch K. Dabiré designed the investigations; SPS coordinated the field collection; Simon P. Sawadogo, Didier A. Kabore and Angela Hughes performed laboratory analysis of samples, Simon P. Sawadogo, Shannon Quek, Ezechiel B. Tibiri and Olivier Gnankine undertake the phylogenetic Analysis; Simon P. Sawadogo, Grant L. Hughes, Hilary Ranson and Roch K. Dabiré wrote the manuscript with the inputs from Didier A. Kabore, Ezechiel B. Tibiri, Angela Hughes, Shannon Quek, Olivier Gnankine and Abdoulaye Diabaté. All authors read and approved the final manuscript.

## CONFLICT OF INTEREST

The authors declare that they have no competing interests.

## Supporting information


**Figure S1.** Single‐nucleotide variant alignment of all sequences from *Anopheles gambiae*, and *Wolbachia* of *Aedes albopictus*.

**Table S1.** Details of mosquito samples from Burkina Faso tested for the presence of Wolbachia at LSTM.

## Data Availability

The data supporting the conclusions of this article are included within the article and its additional files.
