# Supplementary material for: Lack of robust evidence for a Wolbachia infection in Anopheles gambiae from Burkina Faso
Source: Med Vet Entomol. 2022 Jul 25;36(3):301–8. doi: 10.1111/mve.12601 (PMC10053554; doi:10.1111/mve.12601)
Supplement: Supplementary file 2 — Table S1. Details of mosquito samples from Burkina Faso tested for the presence of Wolbachia at LSTM. [file MVE-36-301-s001.pdf]

Supplementary Table 1: Details of mosquito samples from Burkina Faso tested for the presence of Wolbachia at LSTM

**Location**	**Sample Size**	**Collection Date**	**Pyrethroid resistance phe**	**Species**	
VK7	48	Oct-11	Alive	Presumed *An coluzzii*	
VK7	42	Oct-11	Dead	Presumed *An coluzzii*	
VK7	127	Jun-12	Alive	Presumed *An coluzzii*	
VK7	51	Jun-12	Dead	Presumed *An coluzzii*	
Bakaridjan	41	Jul-13	Unexposed	51 % A*n coluzzii*	
Koflande	19	Jul-13	Unexposed	63 % A*n coluzzii*	
Bounouba	26	Jul-13	Unexposed	77 % *An coluzzii*	
Moussoumourou	10	Jul-13	Unexposed	80 % *An coluzzii*	
Tiefora	24	Sep-14	Alive	*Presumed An coluzzii*	
**TOTAL**	**388**				
